# A Framework for Doing Things *in a Good Way*: Insights on Mshiikenh (Freshwater Turtle) Conservation Through Weaving Western Science and Indigenous Knowledge in Whitefish River First Nation

**DOI:** 10.1002/ece3.71431

**Published:** 2025-05-08

**Authors:** Reta Lingrui Meng, Alexis McGregor, Deborah McGregor, Lorrilee McGregor, Keith Nahwegahbow, Patricia Chow‐Fraser

**Affiliations:** ^1^ Department of Biology McMaster University Hamilton Canada; ^2^ Lands Department Whitefish River First Nation Birch Island Canada; ^3^ Faculty of Science University of Calgary Alberta Canada; ^4^ NOSM University Sudbury Canada

**Keywords:** Anishinaabe worldview, first nation, freshwater turtle, indigenous knowledge, species at‐risk management, stewardship

## Abstract

Co‐developed conservation programs for Species At‐Risk, created in partnership between Indigenous Nations and non‐Indigenous researchers, represent a vital shift toward effective species recovery strategies that are culturally respectful and contribute to reconciliation within the natural sciences. By weaving together diverse knowledge systems and prioritizing Indigenous laws, knowledge values, and community priorities, these collaborations aim to restore species at‐risk populations and prevent species extirpation—a task of increasing urgency amid the global biodiversity decline. As similar partnerships gain momentum across Canada, it is critical to reflect on approaches that honor Indigenous perspectives and actively avoid the historical harms associated with colonial research practices on Indigenous lands. This paper draws on insights from a community‐driven species at‐risk conservation initiative at Whitefish River First Nation, or Wiigwaaskingaa (Elder Arthur McGregor baa, 2000), in Northern Mnidoo Gamii (Georgian Bay), Ontario, Canada, where community members and researchers co‐developed a mshiikenh (freshwater turtle) conservation project. We present this paper as a chance to reflect on our iterative collaborative process, its challenges, successes, and key lessons learned. We focus on six key themes for meaningful collaboration: co‐developing project objectives, honoring community priorities, respecting data sovereignty, the journey of learning and unlearning, focusing on a community‐guided trajectory, and promoting tangible outcomes. By highlighting specific examples from Whitefish River First Nation's mshiikenh conservation project, we demonstrate the value of community‐engaged research as a pathway forward for Species At‐Risk conservation in Canada and beyond.

## Graphical abstract

Co‐developed conservation programs for Species At‐Risk, built in collaboration between Indigenous Nations and non‐Indigenous researchers, offer culturally respectful and effective strategies for species recovery while advancing reconciliation in the natural sciences. Drawing on experiences from Whitefish River First Nation's freshwater turtle conservation initiative, this paper identifies six key themes for meaningful collaboration, including honoring Indigenous priorities, respecting data sovereignty, and fostering tangible outcomes. These partnerships exemplify how community‐engaged research can address biodiversity decline while avoiding colonial research practices.

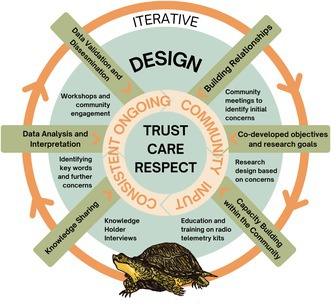

## 1 Introduction

### 1.1 Background and Rationale: High Level Calls for Indigenous Involvement

There are increasing calls for Indigenous involvement in Species At‐Risk conservation at both the Canadian federal and Ontario provincial levels (Hill et al. 2019). This shift is reflected in legislation such as the *Species at Risk Act* and the *Endangered Species Act*, which acknowledge the importance of multiple knowledge systems. However, gaps remain between this official recognition and the meaningful involvement of Indigenous Nations, with over half of all recovery plan documents for species at risk in Canada including no direct statement on the value of Indigenous participation (Hill et al. 2019). Following the Truth and Reconciliation Commission's 94 Calls to Action, Canadian natural scientists are also increasingly urged to re‐evaluate their research methodologies to ensure that Indigenous communities, rights, and knowledge are included and respected in the research process (Wong et al. 2020). Upholding, recognizing, and implementing Indigenous Knowledge (IK) and supporting Indigenous leadership in conservation is also highlighted in Canada's 2030 Nature Strategy as a priority (Environment and Climate Change Canada 2024). Western scientists are encouraged to collaborate with Indigenous peoples to co‐develop programs and management plans, particularly for culturally significant species at risk.

In the context of this paper, co‐development is the collaborative and respectful process in which Western Science researchers and First Nation community members work together to design, implement, and adapt research and conservation initiatives. This approach emphasizes mutual respect, weaving both Western scientific methods and IK, with active involvement from community members in decision‐making, data collection, data analysis, and the sharing of results. Through co‐development, all parties contribute their expertise, ensuring research projects align with the community's values, traditions, and long‐term stewardship goals, while also utilizing scientific methodologies to support conservation outcomes. This collaborative process fosters a deeper, more reciprocal relationship that empowers the community, respects Indigenous sovereignty, and calls for a reimagining of scientific practice grounded in a genuine commitment to valuing all knowledge systems equally. Weaving multiple knowledge systems can help foster collaborative relationships among Western scientists, IK holders, government agencies, First Nation communities, and other stakeholders, thus facilitating evidence‐based Species At‐Risk management. This approach can be conceptualized as a three‐braided system, where IK guides Western Science, grounded in a central strand of respect and reciprocity as the foundation of the knowledge‐weaving process (Kimmerer 2013). In the same way that Western scientific training occurs in classrooms and laboratories, cultural training is equally important. Western scientists can play a key role in breaking down barriers by gaining a deeper understanding of Indigenous traditions, laws, cultures, and nation‐to‐nation distinctions (McGregor 2004). In doing so, we shift from evaluating IK by Western scientific criteria to recognizing each system's distinct strengths and avoiding unintended integration or assimilation (Nadasdy 1999; Reid et al. 2022). Co‐developed and co‐managed species at‐risk recovery planning efforts contribute directly to decolonizing resource management and decentralizing decision‐making from western scientific institutions and governments so that Indigenous rights to self‐determination, treaty rights, and traditional laws are upheld at every critical step (Ignace et al. 2023; Assembly of First Nations 2012).

Canada offers several excellent examples of knowledge weaving, co‐development, and creating ethical space in species‐at‐risk and wildlife management. For instance, *Wah tzee* (i.e., *Rangifer tarandus* , caribou) management on the west coast, led by the West Moberly and Saulteau First Nations, has demonstrated the benefits of Indigenous‐led conservation initiatives, culminating in a Partnership Agreement, a Conservation Agreement under section 11 of the Species At Risk Act, that highlights shared recovery strategy goals among First Nations, provincial, and federal governments (Lamb et al. 2022). In Ontario, collaboration between Western scientists, IK holders, and provincial government agencies has supported *Mooz* (i.e., *Alces alces* , moose) populations through monitoring programs, community‐based interviews, and long‐term relationship building (Yarchuk et al. 2024; Popp et al. 2019; Popp et al. 2020). On the east coast, Mi'gmaw community concerns over the threatened *Gumegwsis* (i.e., *Cyclopterus lumpus* , common lumpfish) led to a co‐developed ecology project aimed at understanding its life history, local significance, habitat threats, and implications for conservation, which contributed directly to the Committee on the Status of Endangered Wildlife in Canada (COSEWIC) documentation on the species (M'sɨt No'gmaq et al. 2024). Within academic institutions, there is a growing recognition of the importance of weaving diverse knowledge systems, exemplified by Nisga'a scientist Dr. Andrea Reid's scholarship on west coast salmon. Reid (2020) utilized the framework of Etuaptmumk, or “Two‐Eyed Seeing,” to provide concrete, on‐the‐ground examples of how IK and Western Science can work collaboratively in conservation and species protection (Reid 2020; Reid et al. 2022, 2024).

These and other examples illustrate the growing body of literature and on‐the‐ground projects focusing on collaborative efforts that promote the meaningful involvement of Indigenous Peoples in Species At‐Risk conservation within Canada, Turtle Island, and beyond.

### 1.2 Freshwater Turtles and Their Status

Applying the concept of weaving knowledge systems is critical for the conservation of culturally significant species, such as freshwater turtles. Mshiikenh (turtles in Anishinaabemowin) have many cultural significances in First Nations communities across Turtle Island (i.e., North America), including ceremonial significance as rattles, featuring in various versions of the Turtle Island Creation Story, symbolizing the Turtle clan, and representing Truth in the Seven Grandfather Teachings—Anishinaabe principles that guide people on how to live a good life. In the grandfather teachings, living conscientiously and slowly, like the turtle, encourages us to value not only the destination but also the journey.

Currently, Ontario is home to eight species of freshwater turtles, all of which are considered at risk, with various provincial and federal designations across Ontario and Canada (Desforges et al. 2022). These turtles face numerous threats, including habitat loss due to land conversion, habitat fragmentation (intact habitats divided into smaller isolated fragments by human activities), and road mortality from vehicular collisions (Stanford et al. 2020). Many of these species, especially semi‐aquatic ones like the Blanding's Turtle ( *Emydoidea blandingii* ), require extensive habitats to meet their life requirements, including wetlands, forests, rock barrens, and anthropogenic landscapes for activities such as mating, foraging, nesting, and overwintering (COSEWIC 2016; COSSARO 2017).

The habitat usage, movement patterns, and population trends of freshwater turtles are of interest to researchers, who seek to understand ecological principles, to provincial and federal agencies that aim to develop management plans, and to Indigenous peoples, who exercise their inherent rights in stewarding traditional territories and supporting Nationhood. Land‐based conservation programs hold particular importance for Indigenous communities, as land is central to community health and is where IK originates, supporting strong and healthy relationships between Indigenous nations and their traditional territories (Mikraszewicz and Richmond 2019). Protecting the turtle also supports the revitalization of land and water stewardship, which is a direct exercise of inherent Indigenous rights. In this way, restoring and protecting the land and its inhabitants serves as a pathway to revitalize and center Indigenous worldviews.

By working together, these diverse perspectives and experiences can contribute to a more comprehensive understanding of best management and conservation practices and prevent the imminent extirpation of many freshwater turtle species.

### 1.3 Whitefish River First Nation

Whitefish River First Nation (WRFN) is part of Anishinabek Nation and serves as a leader and partner in this project. WRFN is a signatory to both the Bond Head Treaties of 1836 and the Robinson Huron Treaty of 1850. The WRFN community is located on the north shore of Lake Huron, bordered by McGregor Bay and Bay of Islands. Most community members reside in the village of Birch Island. Like many other First Nations, the village was relocated from the nearby islands to the mainland after the signing of the Robinson Huron Treaty.

WRFN members actively exercise their treaty rights to fish, hunt, gather medicines, and the community is currently conducting land use planning to develop a land stewardship plan. Increasing and persistent industrial threats in the area have promoted formal stewardship action on reserve and in the traditional territories. WRFN is currently expanding its Lands and Resources department to include stewardship of natural resources. WRFN boasts high biodiversity and a variety of habitats, from coastal cattail marshes and upland peatlands to unique alvar ecosystems with rare vegetation and clay ponds that support populations of Blanding's Turtles, Midland Painted Turtles, Snapping Turtles, Northern Map Turtles, and Eastern Musk Turtles, along with many more species at‐risk. This rich biodiversity coupled with the First Nation's interest in conservation and environmental protection, offers an ideal opportunity to collaborate in assessing the current trends and status of freshwater turtles, their critical habitats, and the threats they face within WRFN's traditional territory. By protecting freshwater turtles and their resident wetlands within WRFN, this initiative helps the community gain valuable insights into species and population health, supports decision‐making, and contributes to community well‐being by promoting youth employment, education, and the exercise of treaty rights through land stewardship.

### 1.4 Background on the Development of Insights

**FIGURE 1:**
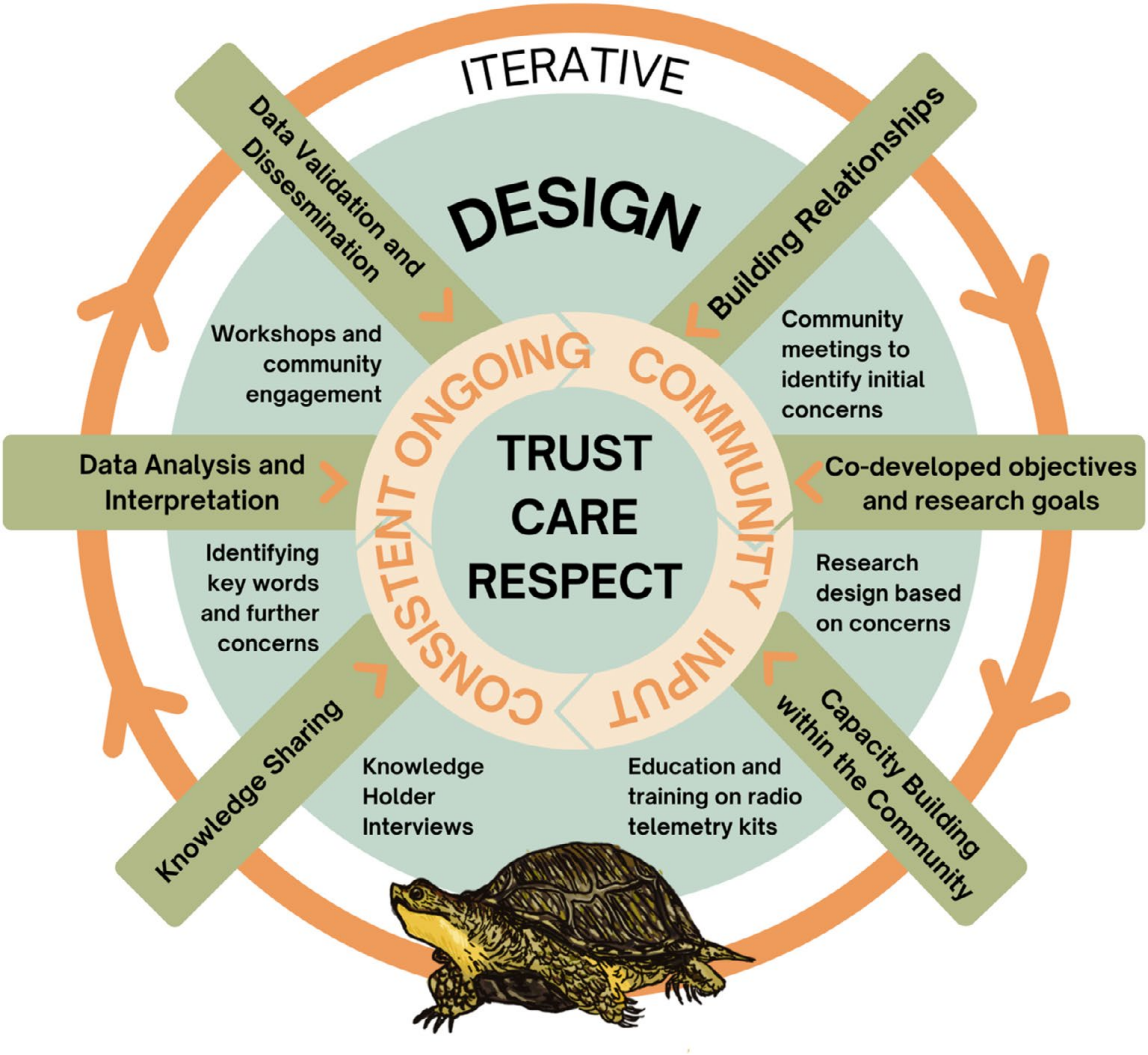
Iterative design framework designed and produced by Reta L. Meng in collaboration with the Whitefish River First Nation Lands and Resources Department; this framework outlines the critical steps in the WRFN mshiikenh conservation program. Community feedback guides each step, and the process is repeated iteratively for each research objective.

We outline six key insights for reflection, drawn from the authors' experiences working together on the WRFN mshiikenh (freshwater turtle) conservation project since 2021. In writing this paper, we did not follow a formal reporting methodology but rather chose to collaboratively reflect on our shared journey, synthesizing the lessons learned and experiences gained throughout the project to offer deeper insights into the collaborative process. This project is a collaborative initiative between the WRFN Lands and Resources Department and McMaster University researchers. It commenced in 2021, following official briefings to the WRFN Chief and Council, which resulted in their approval and support. Since then, annual updates have been provided during council meetings, with additional guidance from Elders and community members to ensure the project aligns with WRFN community objectives and values. The primary goal of the project is to identify, protect, and, where possible, increase freshwater turtle populations within the WRFN reserve and traditional territory. We followed an iterative research framework where community input helps shape each stage of the research program (Figure 1). This goal is being pursued through community engagement, surveys and monitoring efforts, and the identification and mitigation of threats to the turtle populations. In our work, we reflected on conducting research “in a good way,” guided by the Anishinaabe philosophy of *mino bimaadziwin*, which emphasizes that humans live in relation to all our human and non‐human kin (Mikraszewicz and Richmond 2019). In ecological and environmental conservation programming that weaves multiple knowledge systems, the phrase “in a good way” underscores the importance of aligning actions with the specific worldview held by Indigenous partners. It calls for building genuine relationships rather than unequal research partnerships, working with humility, and prioritizing the needs of the community (McGregor 2018). We emphasize that the insights described in this paper are not a prescriptive checklist for Western Science practitioners or Indigenous Nations to follow. Rather, they are a contribution to the broader conversation on reconciling natural sciences through collaborative efforts as seen in recent research (Wong et al. 2020; Hird et al. 2023; Ignace et al. 2023; Kadykalo et al. 2021). This paper aims to provide tangible examples of work conducted “in a good way,” grounded in the central principles of genuine respect, reciprocity, and trust.

## 2 Six Main Insights

### 2.1 Theme One: The Importance of Co‐Developing Objectives

For meaningful environmental stewardship to occur “in a good way,” IK and insight must form the foundation of the program. These ways of knowing are rooted in deep, place‐based relationships with the land, and should guide project development, including project goals, expected outcomes, and funding applications. This approach provides an opportunity to center First Nation voices in land stewardship, creating genuine benefits that support nationhood, build capacity, and contribute to long‐term community goals. True co‐development happens when projects are based on community needs, aligning with community priorities, respecting community protocols and practices, and upholding community values. These priorities are often informed by generations of ecological knowledge, which reflect a reciprocal relationship with the land and its beings. In the context of species‐at‐risk recovery and management, co‐developing objectives can begin with setting monitoring timelines, identifying funding sources, addressing community concerns, designing community engagement, and developing plans of action.

At this stage, research and project priorities can be identified by engaging in accessible, culturally appropriate discussions with community members, chief and council, elders, youth, and department staff (e.g., appropriate honorarium amounts, compensation for transportation costs, hosting in comfortable spaces for elders). The goal at this critical step in co‐development should not be solely focused on obtaining approval via a signed research agreement, but also to build genuine relationships and foster a long‐term commitment to the community. Keeping these objectives in mind, non‐Indigenous researchers and Indigenous peoples alike can recognize their mutual aspirations and the explicit recognition of multiple knowledge systems (e.g., Western Science and Anishinabek knowledge in WRFN), which broadens researchers' worldviews and deepens understandings about species and natural systems of interest.

#### 2.1.1 Implementation in Whitefish River First Nation—Mshiikenh Conservation Program

Initial discussions about the WRFN mshiikenh conservation program began in 2021, driven by the mutual interests of the WRFN Lands and Resources department and McMaster University researchers to develop comprehensive programming aimed at protecting freshwater turtle populations. This initiative also supported the community's goal of stewarding their traditional territories through the protection of land, water, and non‐human relatives based on the Anishinaabe principle of respect in which all things on the land are a gift from the Creator, or mother nature, and humans have a responsibility to steward all these gifts (McGregor 2013). From the outset, this project was guided by the principle that the WRFN community's wealth of Anishinaabe *gikendaasowin* (knowledge) is crucial to the success of the research and that the community's deep understanding of the land and non‐human relatives informs all aspects of the study. This knowledge, passed down through generations and stemming from lived experiences on the land, is fundamental in identifying key habitats, roadkill hotspots, and areas of cultural and ecological significance. This ensures that the study remains place‐based and closely aligned with community values.

During the first 2 years, we worked collaboratively to establish common goals, build allyship, and honor Anishinaabe teachings by conducting field work together, hosting community information sessions, holding formal collaboration meetings, drafting research plans, and applying for funding to conduct field studies. Community members' insights about turtle habitat locations, roadkill sites, and wetlands were central to identifying where to focus the research, and their involvement ensured the research honored these places as living entities rather than just physical locations. We also adhered to the principles of OCAP (Ownership, Control, Access, and Possession) to ensure respectful data sharing, reflecting the community's role as stewards of their territory. We view this as a critical step in building a foundation for the project while advancing our relationships as core project partners who can rely on each other as the research progresses.

After 2 years of discussions and dissemination, we drafted comprehensive short‐term (two‐year) and long‐term (five‐year) programming plans, officially launching the WRFN mshiikenh conservation program in 2023. These plans, which were collaboratively drafted by RM and the WRFN Lands Department, outlined clear goals and objectives, defined roles for both Western science researchers and Indigenous community members, and provided detailed timelines for activities. The plans were submitted to Chief and Council for review, editing, and approval. Based on these plans, we conceptualized an iterative research framework that outlines the critical steps in the project, including building relationships, co‐developing research goals, capacity building, knowledge sharing, data analysis, interpretation, and dissemination. Central to this design is consistent and ongoing community input, grounded in trust, care, and respect, to which we refer back throughout the project (Figure 1). The project's implementation was guided by specific, place‐based stressors and shared research goals focused on: (1) identifying existing critical habitat for protection, (2) mitigating road mortality, and (3) addressing mass nest predation affecting WRFN's freshwater turtles. All subsequent field work was carried out with community field technicians.

All research survey locations were selected with guidance from community members through informal discussions, interviews with knowledge keepers, elder gatherings, and mapping exercises to identify wetlands likely to support turtle populations. Community knowledge played a pivotal role in identifying wetlands likely to support turtle populations, as elders and community members shared their long‐term observations of turtle sightings, population changes, and habitat preferences. This traditional ecological knowledge informed the selection of wetlands, road mortality survey routes, and identified areas where turtles were most likely to be found. For instance, community members shared specific sites where turtles had recently been seen, incidentally captured during trapping season, or recollections of past interactions with turtles from Elders.

This collaborative approach helped pinpoint two primary survey areas in WRFN, thus reducing search time for elusive species like the Blanding's Turtle. In fact, Blanding's turtles and other freshwater species were found in all wetlands identified by the community as areas of interest with high chances of BLTU occupancy. Once turtles were located, we initiated a radio telemetry study on the at‐risk Blanding's Turtle, tagging 21 turtles over 2 years (14 May 2023 and 7 May 2024) to examine movement patterns and home range sizes to map reproductive and overwintering habitat (Meng 2024, unpublished data). This information can assist the community in designating sensitive turtle habitats for protection within the WRFN land stewardship and land use plans, safeguarding these areas from future development. Additionally, this marks the first step in fostering discussions around community education and raising awareness about the importance of these habitats for long‐term conservation, aligning with Anishinaabe teachings about the responsibility to care for the land and all beings.

The second part of the program focuses on identifying road mortality hotspots to guide future mitigation efforts. We conducted road mortality surveys along a 16‐km stretch of Highway 6 that bisects WRFN lands. We conducted these surveys three times a week from May to August in 2023 and 2024 and documented spatiotemporal patterns in road mortality among reptiles, amphibians, and small mammals, with a particular focus on freshwater turtles. We also collected data through participatory mapping exercises, which were guided by community members' intimate knowledge of the land, and helped identify frequent wildlife crossings and roadkill locations. Elders and other community members shared valuable insights on areas where turtles are often observed, and the community's understanding of turtle behavior—such as where they often cross roads during nesting season—was crucial in identifying hotspots. Additionally, we distributed an ESRI Survey123 (an application used for creating and analyzing surveys) form through community newsletters and social media, allowing members to report roadkill sightings. This dataset is now being analyzed for environmental correlates of mortality hotspots to provide the WRFN leadership with data to initiate discussions with provincial agencies regarding steps to reduce road mortality along Highway 6.

The third part of the WRFN mshiikenh conservation program investigates predation rates of freshwater turtle nests along a semi‐decommissioned railroad track on the reserve's western shoreline. During engagement and mapping sessions, community members raised concerns about the frequent sightings of scattered eggshells and the presence of snakes and turtles along the tracks from as far back as the 1950s. Based on this community input, we have been conducting surveys along the entire railroad by foot since 2021 and documenting depredated turtle nests. In response to these concerns and observations, we co‐designed an experiment using quail eggs placed in artificial nests to determine whether predation rates are influenced by the proximity of the nests to railroad tracks and roadways. Had WRFN community members not initially expressed concerns to the Lands and Resources Department based on their extensive knowledge of the land, this long‐term threat to freshwater turtles might have gone unaddressed. These findings will inform potential next steps, including the establishment of an incubation program to protect turtle eggs from predation.

The education component of the WRFN mshiikenh conservation program is also central to its success. We actively seek out partnerships with WRFN's school, daycare, and the WRFN Family Wellbeing department to identify collaborative methods for program delivery that foster a connection to the land and all inhabitants through the focal lens of turtles. So far, we have participated in the WRFN Family Fun Day, powwows, school assemblies, and youth culture camps, and we continue to enhance our capacity to deliver high‐quality mshiikenh‐related programming to WRFN youth to support the further development of their relationship with the natural world. This programming includes hands‐on radio telemetry workshops, short interactive presentations, Q&A sessions, information booths, and community “Track a Turtle” days that has occurred annually since 2023.

The overall success of the WRFN mshiikenh conservation program can be attributed to the strong foundation built during the initial co‐development phase, where Anishinaabe knowledge informed every aspect of the research. By shifting from a model where Western science solely dictates research direction to one where the community shapes the research questions and methods, the project honors the intergenerational knowledge of the WRFN community and respects their role as stewards of the land.

### 2.2 Theme Two: Honoring Community Priorities

We recommend that Western science researchers invest time to understand the socio‐political contexts of different communities and respect their boundaries when working with Indigenous Nations. This includes creating space for Indigenous epistemology, methodologies, and stewardship of traditional territories (McGregor 2017), acknowledging the intimate relationships many Indigenous peoples have with the land, and understanding the crucial cultural connections community members hold with non‐human kin—connections that may differ from the classical Western science perspective of objectively viewing “study species” and the general rule of limiting the anthropomorphization of organisms (Varella 2018).

Discussions should also focus on developing culturally appropriate field and research protocols when working on the land, visiting elders, and educating youth. This requires a shift from a purely Western science, positivist ontology of observing the world through an objective lens—removing oneself from the research project to access the “truth”—to respecting and considering Indigenous worldviews and ontologies that observe “truth” through relationality to the animal, plant, and ecosystem (Luby et al. 2021). For example, if researchers are working with mshiikenh, often viewed as spirited beings in Anishinaabe communities, research and field protocols should honor their spirits as such. This approach allows Western science researchers to uphold Indigenous worldviews and conduct research through a decolonial lens, while supporting and respecting community spiritual and cultural values.

It is important to note that each Indigenous nation varies significantly in terms of traditional laws, worldviews, and governing structures. Western science researchers must recognize these differences and avoid applying a prescriptive formula when engaging with different communities.

#### 2.2.1 Implementation in Whitefish River First Nation—Co‐Developed Animal Use Protocol

In WRFN, we co‐developed culturally sensitive Animal Use Protocols, which helped the non‐Indigenous researcher RM shift their perspective on the study species and approach the work with greater respect. This process involved learning about WRFN's history and community members' relationships with mshiikenh, often regarded as elder siblings in Anishinaabe culture. Community members emphasized that turtles carry valuable lessons through their behavior and interaction with their surroundings. While working on the land, we kept this teaching in mind and learned that by observing the turtles' calm and deliberate movements as they navigate their environment, we can reflect on our own actions—how we move through the world—and the importance of balance, patience, and respect in our lives.

This reflection grounded us in the Anishinaabe principle of reciprocity, which reminds us to return the care and respect we receive from the land and its beings. We came to understand the responsibility to approach fieldwork with humility, recognizing that each encounter with a turtle provided an opportunity to learn more about both the species and ourselves. Throughout this process, we sought to understand and reflect on Anishinaabe worldviews regarding how our actions in the field can both positively and negatively affect animals (Gonzalez et al. 2023). We also engaged in ongoing conversations with WRFN Elders and knowledge keepers to ensure our fieldwork aligned with these teachings and values, reinforcing the importance of acting as respectful stewards of the land.

We conducted semi‐structured interviews with 15 WRFN Elders and land users, asking 10 questions related to turtle habitat, historical sightings, and culturally sensitive methods for working with animals (With approval from WRFN leadership and McMaster University Research Ethics Board ethics clearance #6275). These interviews were collaboratively planned by the WRFN Lands department and RM, through which we received valuable teachings. To maintain confidentiality, we summarized direct quotes into four key teachings: (1) When interacting with turtles, always handle them with respect and address them by their names so that each animal understands our good intentions; (2) When encountering deceased animals, place tobacco down with an appropriate prayer, and return the body to the land when possible to honor its life (e.g., if a carcass is found on concrete pavement, remove it safely and gently place it back into the adjacent wetland or forest); (3) Always ask before taking anything from the land, water, or animals, and only take what you need—never be greedy or abrasive when gathering research data; and (4) Approach each day, each animal, and each wetland with a good heart and good intentions.

The non‐Indigenous researchers involved in this project also made genuine efforts to respect WRFN's customs, ways of knowing, and ways of life, while providing ongoing capacity support where possible. Each mshiikenh we worked with was honored for their unique personality, movement patterns, habitat use, and relationships with other turtles. We greeted them each time we met and, when encountering new turtles, we introduced ourselves and our good intentions, handling them gently and respectfully. We learned to slow down and appreciate the patience each turtle showed us throughout our journey together in the project, respected their space as autonomous beings, and mourned their lives when we witnessed road mortalities. If we harvested anything from the land (e.g., soil samples, egg fragments) to collect data, we made sure to offer Asemaa (tobacco) beforehand to show respect. When animal mortalities occurred, we placed Asemaa down to honor their lives and spoke a prayer learned under the guidance of the Department of Lands and Resources staff and WRFN elders.

By actively participating in these culturally sensitive animal use protocols in addition to the established McMaster University Animal Use Protocol, RM has particularly benefited from a broadened perspective, viewing turtles not just as study organisms but as kin and older, wiser siblings from whom we can learn.

### 2.3 Theme Three: Respecting Knowledge and Data Sovereignty

Western Science researchers working with Indigenous nations can benefit from critically evaluating their participation in the colonial history of the natural sciences. This evaluation includes introspective reflection on personal interests, identifying any potential ulterior motives, engaging in self‐paced learning about historical oppression and the extractive colonial research practices that have been conducted in Indigenous communities, and participating in training that supports cultural literacy and competency before and while working in a community. Genuine relationship‐building and humility are essential at this stage, enabling researchers to adopt an “at‐service” mentality toward the communities they engage with.

When working in a community to which one does not belong, Western science researchers are not simply entering a “study site”; they are engaging with a rich array of cultures, relationships, knowledge, and livelihoods. Researchers are working on land that Indigenous peoples live in relationship with daily, and to which they have inherent rights to steward, self‐determine, and access resources. Therefore, data produced on and from the land need to be returned to the community, be owned by the community, and disseminated within the community.

Knowledge co‐production partnerships should adhere to Indigenous data governance principles, such as the First Nations principles of Ownership, Control, Access, and Possession (OCAP) and the CARE principles (Collective Benefit, Authority to Control, Responsibility, Ethics). These principles ensure that Indigenous nations have the right to self‐determine and self‐govern their data, and they enforce the ethical use of that data. It is critical to continually reflect on these principles and actively work toward true reconciliation and healing these relationships through the respectful weaving of knowledge systems throughout the entire span of the project and beyond.

#### 2.3.1 Implementation in Whitefish River First Nation—Data Sharing Protocol

In WRFN, the role of RM is to be “of service” to the community, providing capacity support where appropriate. Early in the project timeline, RM, PCF, and the WRFN Lands Department collaboratively drafted and signed a formal data‐sharing agreement, which outlines the specific roles, ownership, control, access, and possession of data. This agreement affirms that all data generated within WRFN, including knowledge keeper interviews, turtle relocation data, road mortality data, and nest predation data, are owned by WRFN. The agreement upholds WRFN's rights to self‐determination in how this data is used. External researchers can access the data based on mutual trust and established relationships with WRFN, but WRFN must be informed and provide consent for any sharing of research outcomes. All collected data are securely stored on WRFN's designated drive, with a backup copy stored on a separate hard drive. WRFN is also actively exploring long‐term data storage options to ensure that the community has the necessary capacity to manage and protect this data moving forward.

With aid from these agreements, we identified the benefits of the project for both the community and the water, land, and non‐human relatives, and laid out timelines and expectations. This process helps us set clear boundaries and protect community rights in self‐determining data usage. Rather than maintaining a centralized method of Western Science researchers generating research outcomes, we adopt a holistic view in designing the project to achieve outcomes that benefit both the environment and the community's needs. We focus on developing outreach and educational programming that increases awareness and interest toward the turtle project within WRFN, fosters youth curiosity toward the natural world, and welcome active feedback at each stage of the program. As one elder from WRFN says, “Our goal here at WRFN is to raise the next generation of environmental conservationists.”

To increase financial capacity, we co‐developed funding applications to support programming delivery, the purchase of materials and equipment, and the creation of job postings. Since 2021, we have worked together to create training opportunities (e.g., radio telemetry, hoop net trapping, GIS and remote sensing analysis, and IK and worldview education), provide references for youth pursuing higher education, and maintain relationships beyond research capacities to support the genuine holistic growth of all individuals involved in the project. RM also actively supports a wide array of other WRFN Lands and Resources Department initiatives beyond the turtle project, including Walleye rearing and releasing programs, contamination and decommissioning studies, bat population research, and more.

True reciprocity begins to take shape when external researchers, given the capacity, can support the community's goals beyond the conventionally expected research activities of data collection, analysis, and dissemination. This includes investing in relationality, individual career growth, fostering future generations, and recognizing the privileges and responsibilities that come with being welcomed into a community.

### 2.4 Theme Four: Learning and Unlearning

Non‐Indigenous scholars working with Indigenous partners can greatly benefit from frequently re‐examining their personal beliefs about Indigenous peoples and being proactive in self‐guided learning rather than placing the burden of education on Indigenous scholars and community members (Regan 2010). They can achieve this by showing genuine respect, developing an appropriate level of cultural competency, and practicing humility and openness when learning from and interacting with community members (Porter 2004). When approached with an open heart, the process of unlearning can occur, allowing individuals to critically reexamine their existing perspectives on Eurocentric knowledge and concepts. This reflection process is especially important when non‐Indigenous scholars enter a community. They should understand and honor the complex histories of unique communities and recognize the generational legacies these histories can have on all aspects of a community's well‐being, governance, and research interests. It is critical for everyone involved in co‐development processes to acknowledge and respect the existence and autonomy of multiple knowledge systems, agreeing on the importance of collaboration to build programming. Without a genuine interest in working together from all parties, co‐development risks becoming merely performative.

While mistakes and failures can be daunting, they can also present valuable opportunities in the co‐development journey if all those involved hold good intentions and prioritize the interests of the community. This process can be challenging and will continue to test mindsets. Therefore, it is important for all parties involved in the learning process to prioritize their own holistic well‐being, recognizing the difficulties inherent in the consistent learning and unlearning journey (George 2023; Srigley and Varley 2018).

#### 2.4.1 Implementation in Whitefish River First Nation—Co‐Benefits of Research

In WRFN, we have mobilized interested community members to guide turtle programming from a holistic perspective that considers the well‐being of land, water, turtles, and people. This approach removes the top‐down perspective where Western science researchers take the lead in defining the importance of the research project and promotes a welcoming space for co‐learning. Through discussions, visits with elders, and semi‐structured interviews, we have identified co‐benefits that the community values, including, but not limited to: (1) increasing youth connection with the land, (2) offering career‐building opportunities for youth, (3) supporting increased financial capacity to conduct surveys, build knowledge, and Lands department capacity, (4) contributing to broader initiatives to protect land, water, and non‐human relatives, (5) increased understanding of WRFN's territory and specific habitats, and (6) support for environmental programming and education for community youth, students, and children.

The uniqueness of Mshiikenh, as animals that occupy both land and water, is emphasized here and has prompted us to begin developing both land‐based and water‐based education programs to support WRFN's educational goals. Unlearning has occurred in various ways during this process, with RM learning about the holistic benefits of working with Mshiikenh and recognizing the strong sense of responsibility many WRFN community members hold in honoring their relationship with the natural world by taking care of it. RM has begun to view animals and plants as spirited beings, respecting their autonomy, asking for consent before gathering data (through tobacco offerings under WRFN guidance) and supporting Indigenous‐led education for future generations in the WRFN Shawanoswe school, summer camp programs, and WRFN Family Wellbeing programming.

We continue to engage in conversations about funding, programming, and honoring collaboration through co‐authored papers and fieldwork plans each year, while also recognizing that research programs can overburden communities and remain cognizant of this fact.

### 2.5 Theme Five: Community‐Guided Trajectory

Western scientific research often relies on strict academic timelines dictated by institutional standards and funding agencies. However, it is critical to understand that these rigid timelines can be harmful and counterproductive when imposed on co‐developed programs in Indigenous communities. Building relationships can take years, which is atypical compared to standard research partnerships among non‐Indigenous collaborators in the natural sciences. Trust must be earned by Western science researchers, given the harmful colonial history and exploitative practices that have persisted in natural science research. This aspect of relationality remains largely unrecognized as a significant research accomplishment in most academic institutions.

Community priorities can and often will change based on an array of factors including current sociopolitical dynamics and community events, regardless of existing research agendas. Therefore, it is essential to develop project timelines that align with and respect community priorities. Researchers can support this goal by referring to Theme 1, where co‐developed research objectives and clear expectations established from the inauguration of the project help establish reasonable timelines. This approach supports the long‐term success of programming while acknowledging the inevitable ebbs and flows of community life with changing governance, funding availability, and even global events such as the COVID‐19 pandemic.

Securing long‐term funding that allows for flexibility can be challenging for both researchers and Indigenous communities. Therefore, we urge funding agencies to consider these evolving needs and align funding processes with community capacity to enhance accessibility. For example, one funding program has annual funding deadlines that consistently fall in October, which directly overlaps with many First Nation communities' fall hunting season in Ontario. During a time of low capacity within many Lands and Resources offices, funding agencies should develop a better understanding of the cultural significance of the calendar and alter their timeline accordingly.

#### 2.5.1 Implementation in Whitefish River First Nation—Research Governance

The progression of the WRFN turtle program since 2021 has been guided by support and approval from the WRFN Chief and Council. Accessible platforms are consistently provided for community members to offer feedback on project experimental designs, survey locations, project trajectory, and data analysis and interpretation. When possible, we adhere to community protocols by visiting elders in person and in spaces where they feel comfortable, rather than expecting them to travel to the band office for meetings or virtual calls. To facilitate collaboration, we established the *Mshiikenh Ganawaabanjige* (Anishinaabemowin for “Those Who Watch Over Turtles”) working group, which includes Elders, land users, Chief and Council, Lands Department staff, community youth, and RM as the representing researcher. This group meets bi‐annually to foster a continuous process of co‐learning, appreciation, and listening. During these meetings, we present, analyze, and discuss data and project progress together. The meetings also serve as a space for community members to provide feedback, voice concerns, and guide the ongoing trajectory of the program. In addition, we host community workshops, conduct participatory mapping exercises, and engage the general community in feedback activities on an annual basis. In‐depth interviews with knowledge holders help us understand the Anishinaabe worldview toward Mshiikenh, to better define the co‐benefits of the research to the community, and receive teachings that elders are willing to share.

There have been instances when community events led to the cancelation of research activities, elders disagreed with research methodologies (e.g., researchers did not initially place tobacco down on the land each day as a sign of respect, which was suggested by elders as an important daily protocol when working on the land), and researchers accidentally made culturally insensitive mistakes (e.g., offering tobacco too late when visiting elders, discomfort with recording devices). These situations provide valuable learning opportunities, particularly for RM, highlighting the realities of conducting research with Indigenous communities and learning to take direction from the community, rather than plowing ahead or trying to convince community members of the values of the research activity. However, making mistakes and learning from them often leads to significant growth for all those involved in community‐engaged research. During these mistakes, WRFN community members encouraged RM to learn from them, supporting RM's efforts to acknowledge mishaps and learn from constructive feedback throughout the learning journey. Community members appreciated having agency in the research, allowing them to influence the outcomes and ensuring they are respected in the process. Overall, researchers should be encouraged to recognize the positive impacts of their efforts in building relationships, regardless of the challenges involved.

### 2.6 Theme Six: Promoting Tangible Outcomes

We have navigated the space of co‐development over the past few years and have enabled many tangible outcomes resulting from this process. The research results of co‐developed projects can support renewed First Nation self‐determination in land stewardship, promote continuous learning for all parties involved, and demonstrate to other researchers and government agencies how to conduct work respectfully and effectively. This approach is particularly relevant for managing sensitive and culturally significant species at risk, such as Blanding's turtles, which exhibit varying habitat use depending on their location within their range. Place‐based conservation goals, tailored to different subspecies and subpopulations, that are developed and led by Indigenous communities—who are experts at stewarding their lands and have autonomy over land use planning—can significantly benefit these species (Lamb et al. 2022; Artelle et al. 2021; Fisher et al. 2021).

Furthermore, respecting Indigenous autonomy over traditional territories can help establish networks of nations to collaboratively coordinate land stewardship, honoring each nation's distinct laws and cultures (Turcotte et al. 2021). Ultimately, work conducted in a respectful manner supports direct knowledge mobilization rather than merely discussing co‐production and knowledge weaving as theoretical concepts. The more concrete examples produced through this research that follow these core principles, the more likely it is that conventionally trained western scientists will begin to shift their mindsets.

#### 2.6.1 Theme Six—Looking Forward at Whitefish River First Nation

As of fall 2024, we have completed 2 years of full programming, while the project itself—including the relationship building and planning process—spans 4 years (Table 1). We report our findings and progress to Chief and Council each year and share updates on a biannual basis with our *Mshiikenh Ganawaabanjige* working group. We will continue to build capacity through collaboration with other nearby Anishinaabe First Nations, apply for further funding, and conduct education and outreach initiatives. We have developed co‐authored papers, received continued support from Chief and Council, fostered career growth for youth by providing employment opportunities, and are in the planning stages of developing land‐ and water‐based educational curricula for the WRFN Shawanoswe School in collaboration with the WRFN Education department. We have specifically observed that Blanding's turtles in this area utilize deep, open water for navigation, a habitat not often described in southern populations of Blanding's turtles (Meng and Chow‐Fraser 2023; Meng 2024, unpublished data). Additionally, we have delineated up to 1500 ha of land as BLTU critical habitat within WRFN, which has been reported to Chief and Council and will directly inform land‐use planning within the Nation. The knowledge generated from our program can contribute to SARA recovery strategies, fill gaps in understanding critical habitat requirements, facilitate conversations between WRFN government and provincial government agencies regarding improved highway management, and support WRFN's goals of documenting flora and fauna within their community and traditional territories. By collecting data generated by the Nation for the Nation, we can better support Indigenous self‐determination by addressing community priorities and concerns, ultimately resulting in impactful and tangible outcomes.

**TABLE 1:** Detailed timeline of significant milestones in the Whitefish River First Nation mshiikenh conservation program.

Program milestones	Year
Collaboration began for field work	2021
First community presentation	2022
Formal collaboration meeting	2022
Aboriginal fund for species at risk application	2022
Collaboration on first paper: conservation of freshwater turtles in the anthropocene: indigenous‐engaged approaches to tackle a timeless problem (Meng et al. 2024)	2023
Youth employment	2023
WRFN species booklet creation	2023
First Mshiikenh Ganawaabanjige meeting	2024

## 3 Fostering Indigenous‐Centred and Culturally Sensitive Species At‐Risk Recovery

Indigenous‐led and Indigenous‐engaged conservation initiatives are increasingly gaining momentum across Canada (Indigenous Circle of Experts 2018). However, there remains a gap in providing appropriate cultural and social training for natural science researchers before they engage with Indigenous communities. The WRFN mshiikenh conservation program provides an example of how genuine efforts to ensure cultural competency and build research around community interests can lead to the creation of holistic, long‐lasting conservation plans. Through our experience of weaving IK with Western Science in a co‐production process, we have successfully fostered reciprocal research relationships between university‐affiliated natural scientists and the First Nation community. These relationships can be further developed by the community, allowing for continued collaborative research based on their needs and interests. This collaborative approach not only enhances information sharing from all sources but also fosters a respectful framework for working together. We hope this paper contributes to the broader and vital conversation about honoring and centering Indigenous worldviews in land, water, and wildlife management. We wish to emphasize that the examples shared in this paper are unique to WRFN and, given the vast diversity of IK systems and nation‐to‐nation differences, are not intended as a sole resource for researchers aiming to build successful collaborative relationships. Instead, they offer a set of insights to consider when engaging in the co‐development of research programs.

While the collaborative approach outlined in this paper presents many opportunities, several challenges must also be acknowledged. First, not all Indigenous communities may have the capacity to sustain the level of engagement required for meaningful collaboration, especially without adequate resources or support. Additionally, research advisors often lack the specific knowledge or training needed to guide students in working respectfully and in alignment with Indigenous practices. Another challenge lies within the current structure of many grant applications, which typically require an IK component. However, the timelines of these grants often do not allow for the deep, genuine collaboration needed to ensure the involvement of Indigenous communities in a meaningful way. Furthermore, the expected outcomes of such grants are frequently misaligned with community priorities, preventing authentic, co‐developed projects from taking root. Addressing these existing challenges is essential in advocating for the development of conservation programs that better support and respect Indigenous communities and practices.

Given that much of Canada's biodiversity and at‐risk species are found within Indigenous‐managed lands (Schuster et al. 2019), the development of constructive, reciprocal relationships is crucial to ensuring long‐term conservation and recovery of these populations. As more Indigenous‐led and co‐developed programs are implemented across Canada, a beacon of hope is lit for species at risk from coast to coast.

## Author Contributions

**Reta Lingrui Meng:** conceptualization (lead), data curation (lead), formal analysis (lead), funding acquisition (supporting), investigation (lead), methodology (lead), project administration (equal), visualization (lead), writing – original draft (lead), writing – review and editing (lead). **Alexis McGregor:** conceptualization (supporting), data curation (supporting), investigation (supporting), visualization (supporting), writing – review and editing (supporting). **Deborah McGregor:** conceptualization (supporting), investigation (supporting), supervision (equal), writing – original draft (supporting), writing – review and editing (equal). **Lorrilee McGregor:** conceptualization (supporting), methodology (supporting), supervision (equal), validation (supporting), writing – original draft (supporting), writing – review and editing (equal). **Keith Nahwegahbow:** funding acquisition (lead), investigation (supporting), project administration (equal), resources (supporting), supervision (equal), writing – review and editing (equal). **Patricia Chow‐Fraser:** funding acquisition (lead), supervision (equal), writing – review and editing (equal).

## Conflicts of Interest

The authors declare no conflicts of interest.

## Data Availability

The authors have nothing to report.
